# NAD Metabolism in Acute Myeloid Leukaemia: Biological Rationale and Therapeutic Opportunities

**DOI:** 10.3390/nu18142295

**Published:** 2026-07-13

**Authors:** Klaartje Somers, Mawar Karsa, Donia M. Moujalled

**Affiliations:** 1 Children’s Cancer Institute, Minderoo Children’s Comprehensive Cancer Centre, Sydney, NSW 2031, Australia; ksomers@ccia.org.au (K.S.); mkarsa@ccia.org.au (M.K.); 2 School of Clinical Medicine, UNSW Medicine & Health, UNSW Sydney, Sydney, NSW 2052, Australia; 3 Walter and Eliza Hall Institute of Medical Research, Melbourne, VIC 3052, Australia; 4 Department of Medical Biology, Faculty of Medicine, Dentistry and Health Sciences, University of Melbourne, Melbourne, VIC 3010, Australia; 5 Australian Centre for Blood Diseases, Monash University, Melbourne, VIC 3044, Australia

**Keywords:** NAD metabolism, NAMPT, mitochondrial metabolism, oxidative phosphorylation, metabolic reprogramming, cancer metabolism, acute myeloid leukaemia, NAD supplementation

## Abstract

Acute myeloid leukaemia (AML) exhibits profound metabolic plasticity that enables leukaemic cells to survive environmental stress, nutrient limitation, and therapeutic pressure, ultimately driving disease persistence and relapse. While genetic and epigenetic alterations have guided risk stratification and therapeutic development, accumulating evidence indicates that nutrient-dependent metabolic rewiring represents a critical and targetable vulnerability in AML. Nicotinamide adenine dinucleotide (NAD) is a central metabolic cofactor whose intracellular availability is tightly linked to dietary intake of its precursors, including tryptophan, niacin (vitamin B3), nicotinamide, and nicotinamide riboside. NAD supports redox balance, mitochondrial metabolism, DNA repair, and stress adaptation, processes that are particularly critical for leukaemic stem cell survival under therapeutic stress. Recent studies demonstrate that AML cells, including those resistant to venetoclax- and hypomethylating agent–based regimens, exhibit heightened dependence on the NAD salvage pathway mediated by nicotinamide phosphoribosyltransferase (NAMPT). Pharmacological inhibition of this pathway induces profound NAD depletion, mitochondrial dysfunction, and selective leukaemic cell death. In this review, we integrate nutritional biology with emerging translational evidence to examine NAD metabolism as a nutrient-regulated metabolic vulnerability in AML. We discuss dietary sources and systemic regulation of NAD, the role of NAD-dependent pathways in leukaemic persistence, the translational exploitation of NAD salvage dependency, and the emerging controversy surrounding NAD supplementation in cancer. Finally, we highlight key knowledge gaps and future directions at the interface of nutrition, metabolism, and therapy response in AML.

## 1. Introduction: Metabolic Reprogramming in Acute Myeloid Leukaemia

Acute myeloid leukaemia (AML) is a biologically heterogeneous and clinically aggressive hematological malignancy, characterized by the clonal expansion of immature myeloid progenitors in the peripheral blood and bone marrow, leading to suppression of normal hematopoiesis [1]. Despite advances in genomic classification and the development of targeted therapies, including the BCL-2 inhibitor venetoclax in combination with hypomethylating agents such as azacitidine, relapse remains common and overall survival remains poor, particularly in older patients and those with adverse-risk disease [2].

Historically, AML pathogenesis and treatment resistance have been viewed primarily through the lens of genomic aberrations and epigenetic dysregulation. However, increasing evidence indicates that leukaemic stem cells (LSCs) rely heavily on metabolic reprogramming to sustain proliferation, resist programmed cell death, and adapt to hostile microenvironmental conditions [3]. AML cells reside within the bone marrow niche, an environment characterized by low oxygen tension, limited nutrient availability, and competition with normal hematopoietic cells [4]. These constraints impose selective pressure favoring leukaemic populations capable of metabolic flexibility and efficient nutrient utilization.

Metabolic reprogramming and nutrient addiction are widely recognized hallmarks of cancer. In malignant cells, metabolic pathways are rewired to support rapid proliferation and survival under stress. This reprogramming serves two fundamental functions: the generation of ATP and metabolic intermediates required for the synthesis of nucleotides, amino acids, lipids, and redox cofactors such as nicotinamide adenine dinucleotide phosphate (NADPH), and the modulation of nutrient-sensing signaling pathways that regulate cellular growth and survival [5].

One of the best characterized metabolic phenotypes of tumor cells is the Warburg effect, in which cancer cells preferentially utilize aerobic glycolysis for rapid ATP production rather than relying solely on mitochondrial oxidative phosphorylation (OXPHOS) [6]. However, it is now increasingly recognized that cancer cells exhibit substantial metabolic plasticity, supporting not only rapid proliferation and tumor growth, but also enabling cancer cells to evade therapeutic strategies [7,8,9].

In AML, metabolic pathways that integrate nutrient availability with cellular stress responses have therefore emerged as critical regulators of leukaemic cell survival. Among these, nicotinamide adenine dinucleotide (NAD^+^) metabolism represents a central metabolic hub, linking mitochondrial energetics, redox homeostasis, and stress adaptation [10,11,12], making it an important pathway in AML biology.

Beyond its central role in cellular metabolism, NAD^+^ also occupies a unique position at the interface between intracellular metabolic regulation and systemic nutrient availability. Cellular NAD^+^ pools are maintained through multiple biosynthetic pathways that utilize nutrient-derived precursors, linking dietary intake and whole-body metabolism to intracellular NAD^+^ homeostasis [11]. Consequently, NAD metabolism represents a metabolic axis through which nutrient availability may influence cellular bioenergetics, redox balance, and stress adaptation in both normal and malignant cells.

## 2. NAD as a Nutrient-Derived Metabolite: Dietary Sources and Systemic Regulation

NAD is synthesized through three biosynthetic pathways that utilize nutrient-derived precursors, including tryptophan, niacin (vitamin B3), nicotinamide (NAM), nicotinamide riboside (NR), and nicotinamide mononucleotide (NMN) [11,13,14]. Among these precursors, niacin represents an essential dietary nutrient, with recommended daily intake levels of approximately 14–16 mg/day for adults, reflecting its role in maintaining systemic NAD metabolism [14]. The primary dietary sources of tryptophan include fish, dairy products, nuts, seeds, eggs, bananas, oats, soy, and meat. Additionally, trace amounts of NR and related metabolites are found in vegetables, meat, and milk. These compounds are obtained through diet and supplementation, supporting increases in cellular NAD pools [15]. Dietary intake of NAD precursors varies depending on diet, age, metabolic status, liver function and disease state. In cancer patients, nutrient intake is frequently compromised due to gastrointestinal malfunction, altered absorption, and systemic inflammation [16]. As the principal organ regulating systemic NAD metabolism, the liver coordinates precursor uptake, de novo NAD synthesis, inter-organ precursor distribution, and NAD turnover, thereby influencing systemic NAD precursor availability. These factors may collectively influence intracellular NAD biosynthesis and metabolic fitness. Although these relationships have not been systematically studied in AML, the dependence of NAD metabolism on dietary inputs suggests that nutritional status may influence leukaemic metabolic fitness.

**Figure 1:**
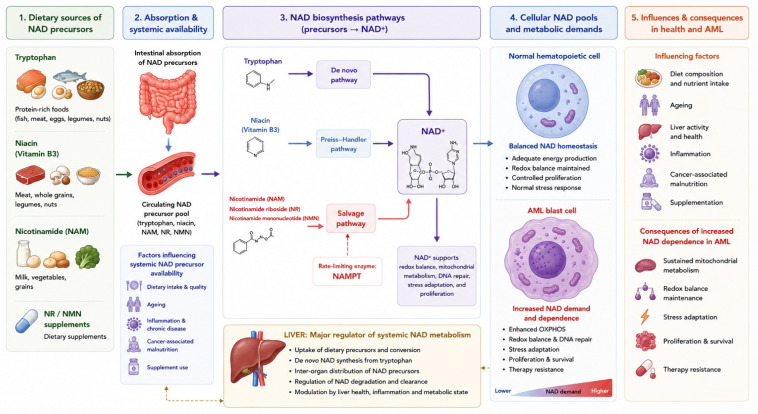
Dietary, biochemical, and systemic regulation of NAD metabolism in health and AML. NAD is synthesised from multiple nutrient-derived precursors through three interconnected biosynthetic pathways: the de novo pathway from tryptophan, the Preiss–Handler pathway from niacin (vitamin B3), and the salvage pathway from nicotinamide (NAM), nicotinamide riboside (NR), or nicotinamide mononucleotide (NMN). Following intestinal absorption, these precursors contribute to the systemic NAD precursor pool, which is influenced by dietary intake and quality, ageing, liver function, inflammation, cancer-associated malnutrition, and supplementation. The liver serves as a major regulator of systemic NAD metabolism by coordinating precursor uptake, conversion, inter-organ distribution, and NAD turnover. Within cells, NAD supports essential processes including mitochondrial metabolism, redox balance, DNA repair, stress adaptation, and proliferation. Compared with normal hematopoietic cells, AML blast cells exhibit increased NAD demand and dependence to sustain oxidative metabolism, cellular stress responses, proliferation, and therapeutic resistance, highlighting NAD metabolism as a potential therapeutic vulnerability in AML. Artificial intelligence-assisted image generation (ChatGPT-5.5, OpenAI) was used in the preparation of this figure, with subsequent editing and scientific curation by the authors.

Beyond dietary intake, intracellular NAD homeostasis is governed by a balance between precursor availability, cellular uptake, biosynthetic enzyme activity, and continuous consumption by NAD-dependent enzymes. Prominent among these are poly ADP-ribose polymerases (PARPs), sirtuins, and other NAD-dependent proteins involved in transcriptional regulation, DNA repair, and cellular stress responses [17,18,19]. Because NAD participates in central metabolic and redox reactions, fluctuations in cellular NAD pools can directly influence metabolic capacity and stress adaptation. In rapidly proliferating malignant cells, NAD demand is further increased to sustain glycolysis, mitochondrial respiration, nucleotide synthesis, and redox buffering [19,20]. As a result, maintaining adequate intracellular NAD levels becomes critical for cancer cell survival, placing increased reliance on efficient NAD biosynthetic pathways. Together, these observations support the concept that NAD metabolism is regulated by both intracellular metabolic demand and systemic nutrient availability. The relationship between dietary NAD precursors, systemic NAD regulation, NAD biosynthesis pathways, and leukaemic NAD demand is summarised in Figure 1.

## 3. NAD Biosynthesis Pathways and Their Utilization in AML

Mammalian cells synthesize NAD through three principal pathways: the de novo pathway (Kynurenine Pathway) from dietary tryptophan, the Preiss–Handler pathway from nicotinic acid, and the salvage pathway, recycled from nicotinamide and related metabolites NAM, NA, NR and NMN [12,13]. The relative contribution of each pathway to intracellular NAD^+^ production varies according to tissue type, nutrient availability and metabolic state. Under physiological conditions, the NAMPT-mediated salvage pathway is the predominant source of intracellular NAD^+^ in mammalian tissues because it efficiently recycles NAD degradation products and requires substantially less energy than de novo synthesis [14]. In contrast, the de novo and Preiss–Handler pathways generally make smaller contributions but can become more important under conditions of altered precursor availability or when salvage pathway activity is impaired [14]. Although the contribution of alternative NAD^+^ biosynthetic pathways has not been systematically defined in AML, the marked depletion of intracellular NAD^+^ following NAMPT inhibition suggests that compensatory flux through the de novo and Preiss–Handler pathways is insufficient to maintain NAD^+^ homeostasis under these conditions [21,22].

Emerging evidence indicates that AML cells exhibit preferential reliance on the NAMPT-mediated NAD salvage pathway to maintain NAD^+^ homeostasis and to sustain metabolic demands associated with rapid proliferation and therapeutic stress [23,24]. This metabolic dependence is driven by the high demand for NAD^+^ to sustain oxidative metabolism, maintain redox homeostasis and support DNA repair during therapeutic stress. Leukaemic stem cells display metabolic features consistent with heightened NAD dependency, including elevated mitochondrial oxidative phosphorylation and enhanced resistance to oxidative stress [10]. Therapeutic stress further amplifies this dependency by increasing DNA damage and oxidative stress, thereby elevating NAD consumption through PARP activation and redox buffering [24]. Under these conditions, efficient NAD salvage becomes essential for leukaemic cell survival.

The rate-limiting enzyme of the salvage pathway, nicotinamide phosphoribosyltransferase (NAMPT), catalyzes the conversion of nicotinamide to nicotinamide mononucleotide, which is subsequently recycled back to NAD [25]. The final step of NAD^+^ biosynthesis is catalysed by the nicotinamide mononucleotide adenylyltransferase (NMNAT) family, which converts nicotinamide mononucleotide (NMN) to NAD^+^ in the salvage pathway and nicotinic acid mononucleotide (NAMN) to nicotinic acid adenine dinucleotide (NAAD) in the Preiss–Handler pathway [14,26]. Three NMNAT isoforms have been identified in humans: NMNAT1 is predominantly nuclear, NMNAT2 is cytoplasmic and Golgi-associated, and NMNAT3 localises primarily to mitochondria, supporting compartment-specific NAD^+^ homeostasis [14,26]. Although evidence in AML remains limited, functional studies have identified NMNAT1 as a metabolic dependency that contributes to maintenance of intracellular NAD^+^ homeostasis [27], whereas recurrent genetic alterations or clearly defined functional roles for NMNAT2 and NMNAT3 in AML have not been reported.

Beyond NAD^+^ biosynthesis, maintenance of intracellular NAD^+^ homeostasis also depends on compartment-specific transport and distribution. NAD^+^ functions within distinct subcellular pools, including the nucleus, cytoplasm and mitochondria, where it supports diverse processes such as oxidative metabolism, redox balance, DNA repair and sirtuin activity [28]. Although the mechanisms regulating NAD^+^ transport between intracellular compartments are not yet fully understood, recent studies have identified the mitochondrial carrier protein SLC25A51 as the principal transporter responsible for importing NAD^+^ across the inner mitochondrial membrane [28,29]. By maintaining mitochondrial NAD^+^ pools, SLC25A51 supports oxidative phosphorylation and mitochondrial metabolism. Emerging evidence suggests that AML cells can upregulate *SLC25A51* expression to sustain mitochondrial NAD^+^ availability, thereby promoting oxidative metabolism, proliferation and leukaemic cell survival [30]. Additionally, disruption of mitochondrial NAD^+^ transport impaired oxidative phosphorylation, reduced mitochondrial respiration and compromised leukaemic cell viability, highlighting the importance of NAD^+^ compartmentalisation in AML metabolism [30]. These findings indicate that, in addition to NAD^+^ biosynthesis, intracellular NAD^+^ transport and compartmentalisation contribute to metabolic adaptation in AML and may represent an additional therapeutic vulnerability. However, the regulation of NAD^+^ transport between other intracellular compartments and its broader role in AML biology remain incompletely understood and warrant further investigation.

## 4. Pharmacological Targeting of NAD Salvage as a Therapeutic Strategy

Given the reliance of leukaemic cells on continuous NAD regeneration through the salvage pathway, pharmacological inhibition of nicotinamide NAMPT has emerged as a promising therapeutic strategy [31]. NAMPT inhibition can induce depletion of intracellular NAD, leading to impaired glycolytic flux, lactate production, and maximum mitochondrial respiratory capacity, eventually promoting energy abrogation, ATP depletion, and apoptotic cell death [31,32,33]. Importantly, leukaemic cells, particularly metabolically active and therapy-resistant populations, appear more sensitive to NAD depletion than normal hematopoietic progenitors [34], attributed to higher NAMPT expression levels, reflecting their heightened metabolic and signaling requirements. This metabolic vulnerability is particularly evident in LSCs, which rely heavily on mitochondrial metabolism to sustain survival and therapy resistance. Metabolic profiling studies have demonstrated that LSCs exhibit elevated dependence on NAD-dependent mitochondrial pathways, rendering them sensitive to disruption of NAD regeneration. Consistent with this, targeting NAD biosynthesis has been shown to impair LSC viability and enhance sensitivity to targeted therapies such as the BCL-2 inhibitor venetoclax [21]. Specifically, metabolic analyses of AML LSCs suggest that disrupting mitochondrial metabolic dependencies may impair therapy-resistant stem cell populations and potentially enhance sensitivity to venetoclax-based therapies [21]. Although the early-generation NAMPT inhibitor, FK866 encountered limitations in rodent safety studies and clinical limitations related to dose-limiting toxicities, including retinal toxicities and thrombocytopenia [35], subsequent studies demonstrated that sensitivity to NAMPT inhibition varies substantially across tumour types. While NAD metabolism is essential in all cells, certain malignancies, including AML, ALL, and Ewing sarcoma, exhibit heightened dependence on continuous NAMPT-mediated NAD regeneration to sustain mitochondrial metabolism and survival [10,36,37], thereby creating a larger therapeutic window for NAMPT-targeted therapies. Consistent with this, preclinical studies from our group demonstrate the next-generation NAMPT inhibitor OT-82 has potent anti-leukaemic efficacy in models of poor-risk ALL [38,39] and venetoclax-resistant AML [40], highlighting NAD salvage as a therapeutic vulnerability.

**Figure 2:**
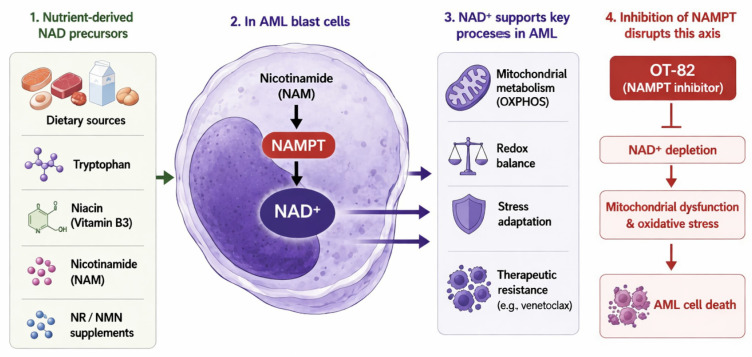
NAMPT-mediated NAD regeneration as a metabolic vulnerability in AML. AML cells rely on nutrient-derived NAD precursors and NAMPT-mediated NAD regeneration to sustain mitochondrial metabolism, redox homeostasis, and survival under therapeutic stress. Increased dependence on oxidative phosphorylation and NAD-dependent metabolic pathways contributes to leukaemic stem cell persistence and resistance to venetoclax-based therapies. Pharmacologic inhibition of NAMPT with OT-82 disrupts NAD^+^ homeostasis, leading to mitochondrial dysfunction, oxidative stress, energetic collapse, and leukaemic cell death, highlighting NAD metabolism as a therapeutically targetable vulnerability in AML. Artificial intelligence-assisted image generation (ChatGPT, OpenAI) was used in the preparation of this figure, with subsequent editing and scientific curation by the authors.

Supporting the translational potential of this strategy, several next-generation NAMPT inhibitors have now advanced into early-phase clinical development. KPT-9274 is currently being evaluated in relapsed/refractory AML (NCT04914845), while RPT1G, a next-generation “hyperbolic” NAMPT inhibitor, has completed Phase I healthy volunteer studies demonstrating safety and target engagement and is planned to enter oncology trials including relapsed/refractory AML and high-risk MDS. Together, these studies highlight renewed clinical interest in targeting NAMPT-dependent malignancies and reinforce the therapeutic relevance of NAD metabolism in AML. From a nutritional and metabolic perspective, NAMPT inhibition represents an acute disruption of a nutrient-dependent metabolic pathway, highlighting the dependence of AML cells on continuous NAD regeneration to sustain metabolic fitness (Figure 2).

## 5. NAD Supplementation, Metabolic Dependency, and Therapeutic Vulnerability in AML

NAD supplementation has gained widespread attention in aging and metabolic health research, driven by evidence that age-associated NAD decline contributes to mitochondrial dysfunction, impaired DNA repair, and metabolic disease [14]. This decline is caused by a combination of reduced biosynthesis and increased NAD consumption by enzymes including CD38, PARPs, and SARM1 [14]. Strategies to restore NAD levels have focused on supplementation with NAD precursors such as nicotinamide riboside (NR) and nicotinamide mononucleotide (NMN), which can be obtained through diet or dietary supplements. Supplementation with these compounds has been shown to increase systemic NAD levels, improve mitochondrial function, and support DNA repair [41]. However, the implications of sustained NAD augmentation in the context of cancer remain controversial. While enhanced NAD availability may support normal tissue homeostasis and stress resilience, malignant cells also exploit NAD-dependent pathways to sustain proliferation, redox balance, and survival under adverse conditions [42]. In AML, where leukaemic cells exhibit pronounced reliance on NAD salvage pathways, increased systemic availability of NAD precursors could theoretically enhance leukaemic metabolic fitness. However, emerging preclinical studies suggest that therapeutic targeting of NAMPT may be tolerated in normal tissues despite profound anti-leukaemic activity. Notably, Korotchkina et al. demonstrated that prolonged treatment with the NAMPT inhibitor OT-82 did not accelerate aging-associated phenotypes in murine models despite sustained anti-leukaemic activity, suggesting that therapeutic NAMPT inhibition may selectively target NAD-dependent malignant cells while preserving normal tissue homeostasis [38].

These observations support the concept of “metabolic addiction,” whereby leukaemic cells become disproportionately reliant on NAD regeneration pathways that are less critical in normal tissues. The concept of metabolic addiction describes cancer cell dependence on specific metabolic pathways that are less critical in normal cells [43]. In AML, heightened reliance on NAD salvage represents a form of metabolic addiction driven by elevated NAD consumption and constrained biosynthetic flexibility. Unlike normal hematopoietic cells, leukaemic cells may lack the capacity to compensate for disruptions in NAD regeneration, rendering them selectively vulnerable to NAD depletion. Within this framework, NAD supplementation and NAD depletion represent opposing forces acting on a shared metabolic axis. While supplementation may alleviate age-related NAD decline in normal tissues, it may simultaneously reinforce metabolic dependencies in malignant cells. This dichotomy highlights the importance of cellular context and disease state when considering nutritional interventions targeting NAD metabolism [14,44]. These implications underscore the need for caution when extrapolating benefits of NAD supplementation observed in aging or metabolic disease to individuals with active malignancy. Indeed, increasing evidence suggests that malignant cells may develop heightened metabolic dependence on NAD availability.

## 6. NAD Metabolism in the Bone Marrow Microenvironment and Anti-Leukaemic Immunity

Although the metabolic dependence of AML cells on NAD has become increasingly recognised, considerably less is known about how NAD metabolism influences interactions between leukaemic cells and the bone marrow microenvironment. Nevertheless, emerging evidence suggests that NAD-dependent processes extend beyond leukaemic cells to influence the bone marrow niche and anti-aemic immune responses, with potential implications for therapeutic efficacy [45]. The bone marrow niche comprises mesenchymal stromal cells, endothelial cells, osteoblasts, adipocytes and immune cells that provide metabolic and trophic support to leukaemic cells [46]. Emerging evidence indicates that NAD metabolism contributes to this bidirectional crosstalk by regulating cellular bioenergetics, redox homeostasis and stress responses within both leukaemic and non-malignant cells [47]. NAD-consuming enzymes, including CD38 and PARPs, are expressed by multiple cell types within the bone marrow and influence extracellular NAD availability, inflammatory signalling and metabolic adaptation [14]. Consequently, therapeutic strategies targeting NAD metabolism may exert effects beyond direct leukaemic cell killing by perturbing the metabolic crosstalk between leukeamic cells and the bone marrow microenvironment [14,48].

NAD metabolism also plays an important role in regulating anti-leukaemic immunity. NAD availability influences the activation, differentiation and function of multiple immune cell populations, including T cells, natural killer cells and macrophages [49], while NAD-consuming enzymes such as CD38 contribute to immune regulation through depletion of extracellular NAD^+^ and modulation of immunosuppressive signalling pathways [50]. Although the effects of NAD-targeted therapies on the immune microenvironment in AML remain incompletely understood, modulation of NAD metabolism may influence both intrinsic leukaemic cell survival and host anti-tumour immune responses. Defining how NAD-targeted therapies affect immune cell function within the bone marrow microenvironment will be important for optimising these agents and identifying rational combination approaches with emerging immunotherapies.

## 7. Nutritional Modulation of NAD Availability as a Therapeutic Adjunct

An emerging but largely unexplored concept is whether nutritional modulation of NAD precursor availability could influence the efficacy of NAD-targeting therapies. Dietary modulation of tryptophan or niacin intake could, in theory, reduce baseline NAD availability and further constrain NAD biosynthesis in leukaemic cells, thereby sensitising them to pharmacologic NAMPT inhibition. However, sensitivity to NAMPT inhibition may also depend on the capacity of leukaemic cells to utilise alternative NAD biosynthesis pathways. Expression of nicotinic acid phosphoribosyltransferase (NAPRT1), the key enzyme of the Preiss–Handler pathway, may provide a bypass mechanism that sustains NAD production during NAMPT inhibition. Consequently, tumour-specific differences in NAPRT1 expression or precursor availability could modulate resistance to NAMPT-targeted therapies [51].

Importantly, this concept remains speculative and must be approached with caution. NAD is essential for normal cellular function, immune competence, and tissue repair, and indiscriminate NAD depletion could result in unacceptable toxicity. Nonetheless, transient or context-specific nutritional modulation, in combination with targeted pharmacologic inhibition, may offer a means to widen the therapeutic window of NAD-targeting strategies, consistent with preclinical studies demonstrating selective anti-leukaemic activity of next-generation NAMPT inhibitors despite preservation of normal tissue homeostasis [38]. Supporting this concept, recent studies in neuroendocrine carcinoma demonstrated that niacin restriction enhances sensitivity to NAMPT inhibition by limiting compensatory NAD biosynthesis through the Preiss–Handler pathway [52]. Similar mechanisms may influence therapeutic responses in AML and warrant further investigation in the context of nutritional modulation and NAD-targeted therapies.

## 8. Future Directions and Knowledge Gaps

Future research should aim to better define how dietary intake of NAD precursors influences intracellular NAD metabolism in AML, particularly within the bone marrow niche. Although NAD biosynthetic pathways are well characterized, it remains unclear to what extent systemic nutrient availability translates into changes in NAD pools in leukaemic versus normal hematopoietic cells. This question is especially relevant in AML patients, who frequently experience altered nutritional status during therapy. Integrating dietary assessment with metabolic profiling of blood and bone marrow samples may help clarify whether nutritional factors modulate NAD-dependent metabolic vulnerabilities in vivo. Importantly, future studies should prioritise physiologically relevant in vivo models, as concentrations of NAD precursors in standard culture media may not accurately reflect systemic nutrient availability or tissue-specific NAD metabolism. Careful consideration of dietary niacin content and systemic precursor availability in preclinical animal studies will therefore be essential. In particular, the contribution of alternative NAD biosynthesis pathways, including NAPRT1-mediated nicotinic acid utilisation, remains poorly defined in AML and may represent an additional determinant of resistance to NAMPT inhibition. In parallel, additional work is needed to determine how nutritional NAD modulation intersects with therapeutic targeting of NAD metabolism. While pharmacologic inhibition of NAMPT has revealed a clear metabolic dependency in AML, the impact of NAD precursor supplementation or dietary modulation on treatment response remains poorly understood. Additional studies integrating metabolomic profiling with genomic analyses may help identify biomarkers of response to NAMPT-targeted therapies and determine whether metabolic states influence therapeutic sensitivity in AML. Addressing this knowledge gap will be important for guiding safe nutritional practices in patients with AML and for evaluating whether nutritional context can be leveraged to enhance the efficacy of NAD-targeting therapies.

## 9. Conclusions

NAD metabolism has emerged as a compelling nutrient-regulated metabolic vulnerability in acute myeloid leukaemia that links systemic nutrient availability with intracellular pathways essential for leukaemic cell survival. Accumulating evidence indicates that AML cells, particularly therapy-resistant and leukaemic stem cell populations, exhibit heightened dependence on NAMPT-mediated NAD salvage to maintain mitochondrial function, redox homeostasis, and adaptation to therapeutic stress. This dependence provides a strong biological rationale for therapeutic strategies that disrupt NAD biosynthesis. In our view, the greatest opportunity for future research lies not only in further developing NAMPT-targeted therapies, but also in understanding how nutritional status and dietary NAD precursor availability influence metabolic dependency and therapeutic response. Integrating nutritional assessment with metabolic profiling and translational models will be essential to identify patients most likely to benefit from NAD-targeted approaches and to determine whether nutritional interventions can be safely incorporated into treatment strategies. Overall, we propose that considering NAD metabolism through both metabolic and nutritional perspectives provides a more comprehensive framework for understanding AML biology and for optimizing future therapeutic strategies. As the field advances, defining the interaction between diet, systemic NAD availability, and aemic metabolic dependencies will be critical for translating NAD-targeted therapies into clinical practice.

## Data Availability

No new data were created or analyzed in this study. Data sharing is not applicable to this article.
